# Integrated Transcriptomics and Nontargeted Metabolomics Analysis Reveal Key Metabolic Pathways in *Ganoderma lucidum* in Response to Ethylene

**DOI:** 10.3390/jof8050456

**Published:** 2022-04-28

**Authors:** Li Meng, Ruyue Zhou, Jialong Lin, Qingji Wang, Panmeng Wang, Wei Wang, Li Wang, Zhuang Li

**Affiliations:** Shandong Provincial Key Laboratory of Agricultural Microbiology, College of Plant Protection, Shandong Agricultural University, Tai’an 271018, China; mengli@sdau.edu.cn (L.M.); 2021120179@sdau.edu.cn (R.Z.); linjialong5e@gmail.com (J.L.); ericwong@sdau.edu.cn (Q.W.); wangpanmeng@mail.kib.ac.cn (P.W.); uniwangwei@sdau.edu.cn (W.W.)

**Keywords:** ethylene, medical fungi, secondary metabolite, polyamine metabolism, ACC pathway

## Abstract

Ganoderic acid (GA) is an important secondary metabolite of *Ganoderma lucidum* with a diverse array of pharmacological properties. In this study, we found that exogenous ethylene increased the production of endogenous ethylene and ganoderic acid in *G. lucidum*. However, the mechanism by which ethylene is regulated remains unclear. As a result, we performed a combined transcriptomics and nontargeted metabolomics analysis to evaluate the regulatory mechanism of ethylene. A total of 4070 differentially expressed genes (1835 up-regulated and 2235 down-regulated) and 378 differentially accumulated metabolites (289 up-regulated and 89 down-regulated) were identified in all groups. The transcriptomics and nontargeted metabolomics data revealed that genes involved in the tricarboxylic acid (TCA) cycle, polyamine metabolic pathway, acetyl-CoA carboxylase (ACC) pathway, and triterpenoid metabolism were up-regulated, whereas the metabolic intermediates involved in these metabolic pathways were down-regulated. These findings imply that ethylene potentially accelerates normal glucose metabolism, hence increasing the number of intermediates available for downstream biological processes, including polyamine metabolism, ethylene synthesis pathway, and ganoderic acid biosynthesis. The findings will contribute significantly to our understanding of secondary metabolites biosynthesis in fungi.

## 1. Introduction

Fungi are prominent organisms that rapidly produce a diverse array of secondary metabolites that protect against UV damage and bacterial ingress, as well as regulate fungal development [1]. A considerable number of secondary metabolites are beneficial to humanity. For instance, ganoderic acid (GA), an important secondary metabolite of *Ganoderma lucidum*, has been shown to contribute to a variety of biological functions, including anticancer [2], antioxidant [3], and liver protection effects [4]. Notably, the biosynthesis of secondary metabolites is regulated by various environmental factors [5], including biochemical signals [6,7,8], physical triggers [9,10], and nutritional conditions [11]. Ethylene has been shown to enhance GA biosynthesis in *G. lucidum* mycelia [12]. However, its regulatory mechanisms remain unknown.

Ethylene is a key gaseous hormone and signaling molecule synthesized by plants, fungi, and bacteria [13]. Ethylene is thought to play an important role in both development and stress responses since it can regulate growth, tissue differentiation, maturation, and responses to numerous stresses. Additionally, ethylene is critical for secondary metabolite accumulation in plants [14,15,16]. Ethylene biosynthesis and signal transduction pathways in the model plant *Arabidopsis thaliana* are well-characterized [17]. Studies indicate that it modulates mushroom postharvest [18], mycelial growth, and primordia formation in *Agaricus bisporus* [19]. However, no consensus on its function within the ethylene-producing fungi has been reached [20]. 

In this study, we combined metabolomics and transcriptomics approaches to assess the potential mechanism of ethylene-regulating GA biosynthesis. These findings will contribute to our understanding of the regulatory mechanism of ethylene signaling in fungi.

## 2. Materials and Methods

### 2.1. Strains and Culture Conditions

A *G. lucidum* strain (accession number: ACCC53264) was provided by Prof. Mingwen Zhao from the Nanjing Agricultural University and preserved at the Agricultural Culture Collection of China. It was incubated at 28 °C on potato dextrose agar (PDA) medium (PDA: potato, 200 g; dextrose, 20 g; agar, 20 g; deionized water, 1 L) for 7 d, and the spawn was prepared in polypropylene bags and incubated at growth conditions similar to those reported in a previous study [21]. Briefly, the inoculated substrate was incubated at 28 °C for mycelial colonization. The air temperature in the cultivation room was maintained at 28 °C and relative humidity at 80–85%. The bags were sprayed with 20 mL of ethephon (15 mM) during the primordial period. Control bags were sprayed with distilled water. The primordia were collected after 24 h of treatment. We collected the fresh primordia of *G. lucidum* and divided them into three portions. One portion of fresh sample was placed in a 10 mL jar and incubated at 25 °C for 24 h, after which it used to determine the ethylene production; one portion of fresh sample was put into liquid nitrogen and was used for RNA isolation and transcriptomics and metabolomics analysis; and one portion of fresh sample was dried at 60 °C and used to determine the ganoderic acid content. 

### 2.2. Measurement of Ethylene Production and GA Content

A one-gram fresh sample of primordia was placed in a 10 mL jar and incubated at 25 °C for 24 h. To determine the ethylene concentration, one milliliter of the headspace gas was injected with an air-tight syringe into the GC-9A gas chromatograph (Shimadzu, Kyoto, Japan) equipped with a GDX-502 column and a flame ionization detector (FID). GC parameters were set as follows: temperatures of the column and detector were 70 °C and 140 °C, respectively; and the flow rate of the carrier gas was 40 mL/min. 

GA was extracted from primordia and quantified using a previously described method [22]. One-way ANOVA was used to determine statistically significant differences between samples with IBM SPSS Statistics 20. Differences between samples were considered statistically significant at *p* < 0.001.

### 2.3. Sample Preparation and LC-MS Analysis

A total of 80 mg of each liquid-nitrogen-frozen sample was weighed and transferred to an Eppendorf tube. The sample was extracted using 1 mL of 70% methanol in water. Subsequently, two 5 mm steel beads were added to the solution and processed for 2 min at 60 Hz in an automatic sample fast grinding machine Wonbio-E (Shanghai, China). After 30 min of ultrasonic extraction in an ice-water bath, the extracts were stored overnight at −20 °C. The extract was centrifuged at 12,000× *g* for 10 min. The supernatants were collected, filtered using a 0.22 µm polyvinylidene fluoride membrane, and stored at −80 °C until LC-MS analysis was performed. Twenty microliters of 2-chloro-L-phenylalanine (0.3 mg/mL methanol), as an internal standard, was added to all sample extracts. 

To identify primordial metabolites, 12 samples were randomly selected from three independent biological replicates and analyzed using LC-MS analysis. The metabolic profile was analyzed in both ESI-positive and ESI-negative ion modes using an ACQUITY UPLC I-Class system (Waters Corporation, Milford, MA, USA) coupled with VION IMS QTOF mass spectrometer (Waters Corporation, Milford, MA, USA). The samples were separated chromatographically using an ACQUITY UPLC HSS T3 column (2.1 mm × 100 mm, 1.8 μm) equipped with a binary solvent system (solvent A: 0.1% formic acid in deionized water; solvent B: 0.1% formic acid in acetonitrile). The gradient elution procedure was used: 0–2 min, 5% B; 2–4 min, 5% B; 4–8 min, 30% B; 8–10 min, 50% B; 10–14 min, 80% B; 14–15 min, 100% B; 15.1 min, 5% B; and 16 min, 5% B. The flow rate was 0.35 mL/min, the injection volume was 2 μL, and the column temperature was maintained at 45 °C. The following instrument settings were used: ion source: ESI; capillary temperature: 320 °C; spray voltages: (+3.8, −3) kV; mass scan range: 100–1200; resolution (full scan): 70,000; resolution (HCD MS/MS scans): 17,500; sheath gas flow rate (Arb): 35 (positive ion) and 35 (negative ion); and aux. gas flow rate (Arb): 8 (positive ion) and 8 (negative ion). Metabolomics data were deposited into the EMBL-EBI MetaboLights database [23].

### 2.4. Metabolite Identification and Quantification

To identify metabolites with a differential response, raw data were collected using the software UNIFI 1.8.1. Baseline filtration, peak identification, peak alignment, peak filling, retention time (RT), and normalization of the raw data were subjected to statistical analysis using QI v2.3 (Waters Corporation, Milford, MA, USA). Metabolite identifications were performed using exact mass-to-charge ratios (*m/z*), isotope distributions, fragmentation patterns, and database hits (The Human Metabolome Database, Lipidmaps, and METLIN). Additionally, metabolite identification was performed using a self-written R package and an in-house self-built secondary mass spectrometry database containing 550 metabolites. The following data processing parameters were used: precursor tolerance: 5 ppm, fragment tolerance: 10 ppm, and product ion threshold: 5%. For each condition, compounds missing more than 50% of the values were removed, and the remaining missing values were substituted with half of the minimum value. Qualitative data were assessed using the qualitative outcomes score. Compounds with a score greater than 36 (a full score of 60) were accepted, whereas those with less than 36 were deleted. The score was calculated as follows: 20 points for MS/MS matching, 20 points for MS/MS fragmentation matching, and 20 points for isotopic distribution matching, with a maximum total score of 60 points. Additionally, mixtures of 12 samples with equivalent quantities were used as quality control (QC) samples, and the QC samples were injected to monitor the stability of the analysis. Differentially expressed metabolites in the PR-H_2_O and PR-C_2_H_4_ medium were chosen using a statistically significant threshold of variable influence on projection (VIP) values. *p*-values were obtained from a double-tailed Student’s *t*-test on normalized peak areas. Additionally, the R2Y, Q2Y, and 200-permutation tests were used to assess the quality of the orthogonal partial least squares–discriminant analysis (OPLS-DA) mode. Metabolites with VIP values greater than 1.0 and *p*-values less than 0.05 were considered indicators of differential metabolites. The volcano plot was used to visualize the *p*-value and fold change value, which is useful for differential screening. The MetaboAnalyst 3.0 software was used to construct heat maps.

### 2.5. Transcriptome Sequencing Analysis

Total RNA per liquid-nitrogen-frozen sample was extracted using the TRIzol reagent according to the manufacturer’s protocol. RNA purity and quantification were evaluated using the NanoDrop 2000 spectrophotometer (Thermo Scientific, Waltham, MA, USA). RNA integrity was assessed using the Agilent 2100 Bioanalyzer (Agilent Technologies, Santa Clara, CA, USA). A total amount of 1.5 µg RNA per sample was used as input for RNA sample preparation. All mRNA was broken into short fragments and reversed to cDNA. The cDNA fragments were purified and ligated to sequencing adapters. Following agarose gel electrophoresis and extraction of cDNA from gels, the cDNA fragments with the lengths of 300 bp were purified and enriched by PCR to construct the final cDNA library (primordia with distilled water treatment, and primordia with ethylene treatment). Sequencing libraries were generated using NEBNext^®^ Ultra™ RNA Library Prep Kit for Illumina^®^ (NEB, Ipswich, MA, USA). Clustering the index-coded samples was performed on a cBot Cluster Generation System using TruSeq PE Cluster Kit v3-cBot-HS (Illumina), following the manufacturer’s instructions. After cluster generation, library preparations were sequenced on the Illumina Hiseq platform to obtain 150 bp paired-end reads. About 47 M raw reads for each sample were generated.

The raw data (raw reads) in fastq format were processed through in-house Perl scripts. Clean data (clean reads) were obtained by removing reads containing adapters and poly-N and low-quality reads from raw data. Simultaneously, Q20, Q30, GC-content, and sequence duplication levels of the clean data were calculated. Each downstream analysis was performed using clean and high-quality data. The transcriptome was assembled based on the reads mapped to the reference genome of *G. lucidum* (Project accession number PRJNA71455) [24]. The abundances are reported as normalized fragments per kb of transcript per million mapped reads. A gene is considered significantly differentially expressed if its expression differs between two samples with a fold change > 2 and a *p*-value < 0.05.

The following databases were used to annotate gene function: Nr (NCBI non-redundant protein sequences); Nt (NCBI non-redundant nucleotide sequences); Pfam (protein family); KOG/COG (Clusters of Orthologous Groups of Proteins); Swiss-Prot (a manually annotated and reviewed protein sequence database); KO (KEGG Ortholog database); and GO (Gene Ontology).

### 2.6. Integrative Analysis of Metabolome and Transcriptome

Pearson correlation coefficients were performed to assess the integration off metabolome and transcriptome data. Pearson correlation coefficients were determined using differential gene expression and metabolite concentration data in R, and then cluster analysis heat maps were drawn. Differentially expressed genes and differential metabolites were mapped to the KEGG database. Metabolome and transcriptome relationships were visualized and interpreted using Cytoscape (version 3.4.0) with MetScape plug-in (version 3.1.3).

### 2.7. WGCNA

The weighted correlation network analysis (WGCNA) algorithm in R/Bioconductor was used to identify gene coexpression modules. In constructing the weighted gene network, a soft thresholding power was selected based on the approximate free topology as previously described. WGCNA quantified module membership for each gene as the correlation between the module gene and its associated expression profile. Correlations were established between gene expression modules, abundant microbial taxa, and metabolites from the same samples.

## 3. Results

### 3.1. The Metabolome Profiling of G. lucidum in Response to Ethylene

**Figure 1:**
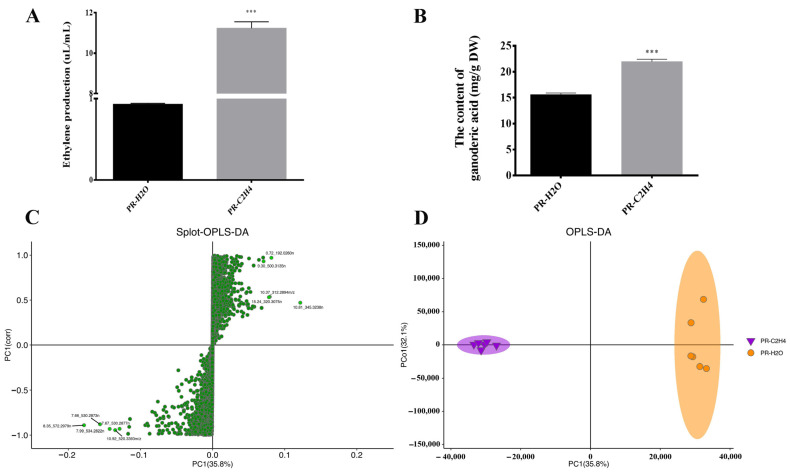
Overview of ethylene production and metabolites of *G. lucidum.* (**A**,**B**) are the ethylene production and the content of ganoderic acid of *G. lucidum*. The histograms marked with *** represent significant differences according to ANOVAs; it indicates statistical significance (*p* < 0.001) compared to the control. PR-H_2_O and PR-C_2_H_4_ represent the primordia of *G. lucidum* treated with water and ethylene, respectively. (**C**,**D**) are the Splot-OPLS-DA and OPLS-DA analysis of different samples.

The results indicate that ethephon significantly increased ethylene production and GA content in *G. lucidum* primordia. The ethylene production increased to 11.23 µL/mL after spraying ethephon on the primordia (Figure 1A). The total GA content after 24 h was 21.96 mg/g, a significant increase of 41% compared to the control (15.59 mg/g) (Figure 1B). Although the findings indicate that short-term treatment with ethylene could improve GA content, the mechanism by which ethylene regulates GA levels remains unexplained. 

The metabolome analysis was performed to have a better understanding of the likely mechanism of GA biosynthesis induced by ethylene treatment. To compare the metabolite compositions of *G. lucidum* primordia treated with ethylene (PR-C_2_H_4_) and the control (PR-H_2_O), LC-MS analysis was performed on primordia flesh samples 24 h following treatment. 

**Figure 2:**
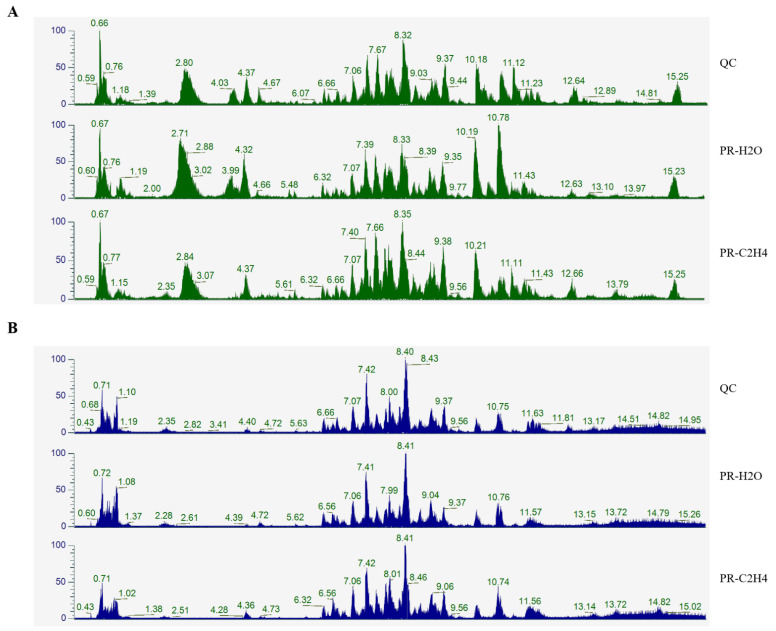
The base peak chromatogram of metabolites. (**A**) Positive ion; (**B**) negative ion. QC, quality control sample; the QC sample was generated from a mixture of equivalent quantities of the 12 samples in this study; PR-C_2_H_4_ and PR-H_2_O represent the primordia of *G. lucidum* treated with ethylene and water, respectively.

The reproducibility of metabolite detection was determined by analyzing the base peak chromatograms using the quality control samples. As shown in Figure 2, the retention periods and peak intensities of metabolites were consistent between quality control and experimental samples, showing that the signal and instrument were stable in our experiment, providing repeatability and reliability for the metabolomics data analysis. Furthermore, orthogonal partial least squares–discriminant analysis (OPLS-DA) was performed to identify differential metabolites, demonstrating that the model was valuable and can be used to screen differential metabolites based on their VIP (Figure 1C). The sample repetition correlation graph demonstrated favorable sample reproducibility, and the metabolome data could be used for subsequent analysis (Figure 1D). Metabolomics data were deposited under the identifier MTBLS3577 in the EMBL-EBI MetaboLights database. The complete dataset can be accessed at https://www.ebi.ac.uk/metabolights/MTBLS3577 (accessed on 7 October 2021).

### 3.2. Differentially Accumulated Metabolites in Response to Ethylene

**Figure 3:**
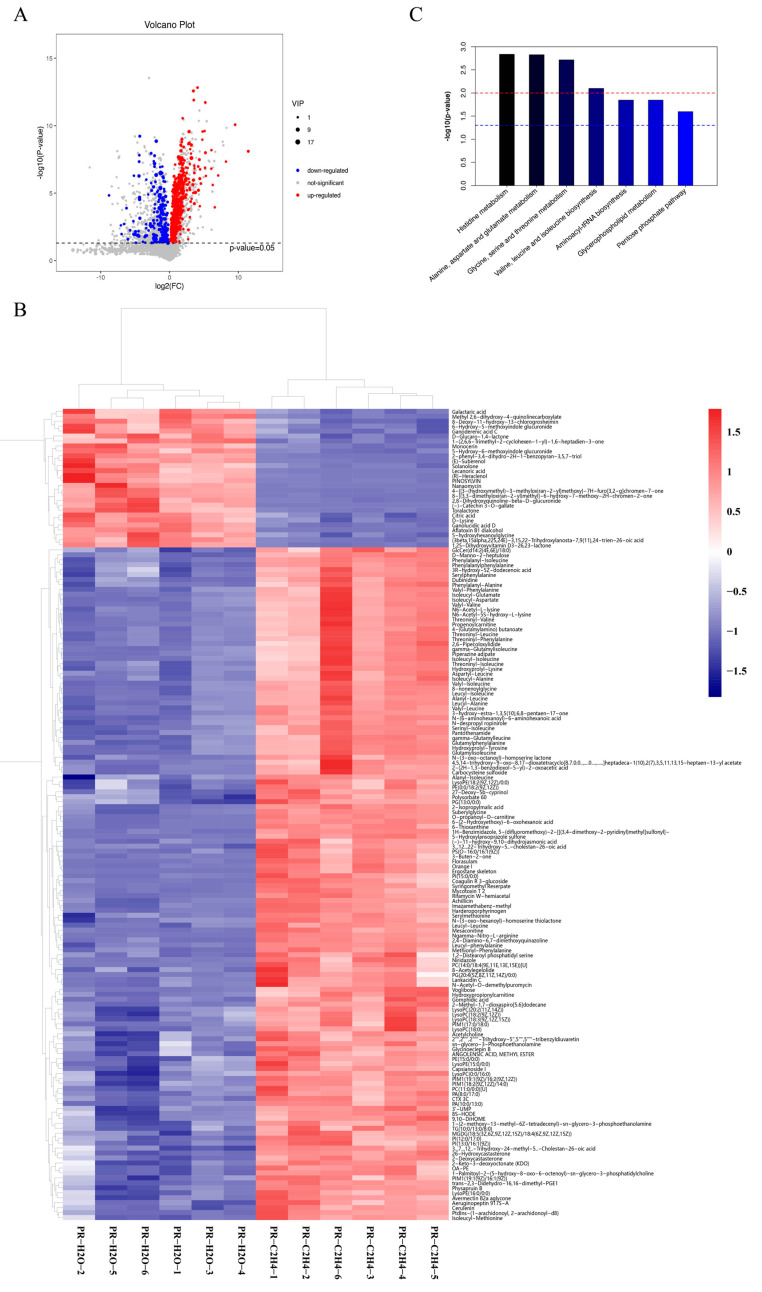
**The differentially accumulated metabolites in response to ethylene**. (**A**,**B**) are volcano plots and clustering heat map of differentially accumulated metabolites. The metabolite content data were normalized. PR-C_2_H_4_ and PR-H_2_O represent the primordia of *G. lucidum* treated with ethylene and water, respectively. (**C**)The most significant 7 KEGG pathways. KEGG is a pathway-related database, and pathway enrichment analysis identifies significantly enriched pathway in differentially accumulated metabolites, and hypergeometric test is used test the statistical significance of the enrichment of differentially accumulated metabolites in KEGG pathways. The red and blue dashed lines indicate *p*-value < 0.01 and *p*-value < 0.05, respectively.

A total of 378 differentially accumulated metabolites were detected in the samples, as shown by volcano plots in Figure 3A, illustrating the significant differences between PR-C_2_H_4_ and PR-H_2_O. A total 289 metabolites were up-regulated, whereas 89 metabolites down-regulated (Table S1). A hierarchical heatmap clustering analysis of the samples revealed that all the biological replicates clustered together, confirming the high quality and reliability of metabolome data (Figure 3B). In addition, the top 20 differentiated metabolites are available in the Supplementary Material, Figure S1. The cluster heat map revealed significant differences in the metabolite profiles of samples treated with ethylene. The differentially accumulated metabolites were grouped into seven classes in the KEGG database. Significantly enriched terms included histidine metabolism, alanine, aspartate and glutamate metabolism, glycine, serine and threonine metabolism, valine, leucine, and isoleucine biosynthesis. Furthermore, the terms were enriched in aminoacyl-tRNA biosynthesis, glycerophospholipid metabolism, and the pentose phosphate pathway (Figure 3C).

Surprisingly, 18 differentially accumulated secondary metabolites were identified in response to ethylene treatment (Figure S2, Table S2). They primarily comprised C27 lanostanes (lucidenic acids) and C30 lanostanes (ganoderic acids). The findings indicate that all metabolites of C27 lanostanes and four metabolites of C30 lanostanes were up-regulated, whereas eight metabolites of C30 lanostanes were down-regulated. These findings suggest that ethylene could help increase the total GA content (Figure 1B).

### 3.3. Transcriptome Profiling of G. lucidum in Response to Ethylene

**Figure 4:**
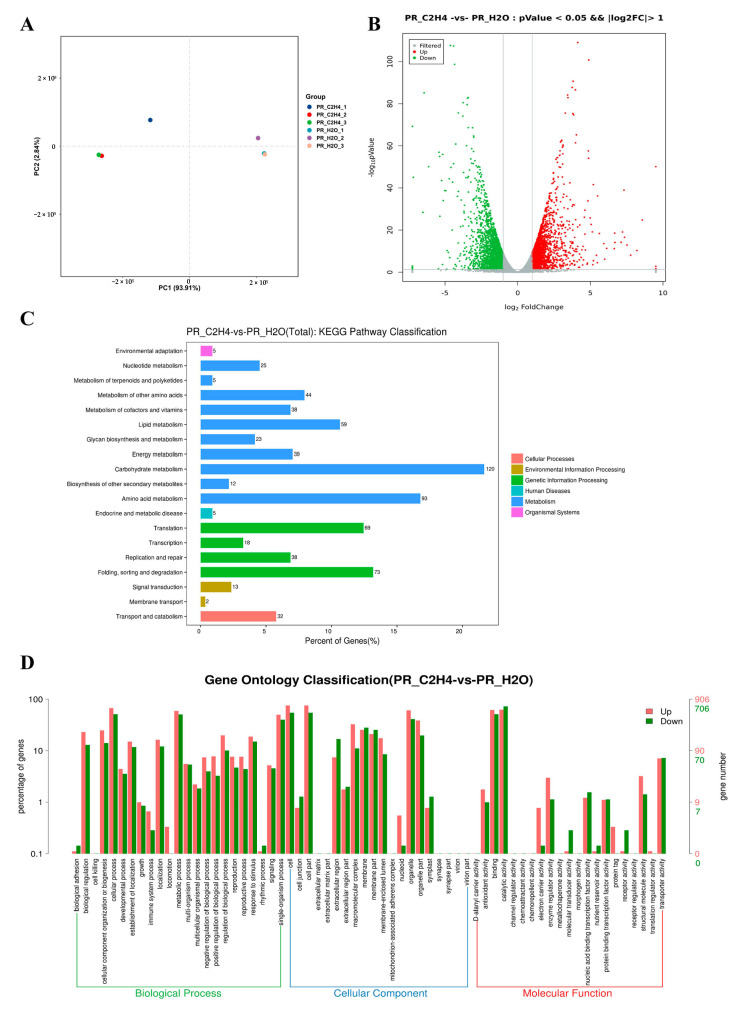
**Qualitative and quantitative analysis of transcriptomics data**. (**A**) The principal component analysis (PCA) of different samples. PR-C_2_H_4_-1, PR-C_2_H_4_-2, and PR-C_2_H_4_-3 are the three samples of primordia that underwent ethylene treatment. PR-H_2_O-1, PR-H_2_O-2, and PR-H_2_O-3 are three control samples that underwent water treatment. (**B**) The volcano plot of differentially expressed genes. (**C**,**D**) are KEGG pathway classification and Gene Ontology classification of differentially expressed genes.

The aggregation correlation of three biological replicates for each sample indicated the strong reliability of the generated transcriptomics data and the large effects of ethylene on genes (Figure 4A). The quality validation results confirm that the transcriptomics data were suitable for subsequent analyses. Transcriptome data were deposited in the NCBI database with the accession number PRJNA769204. The complete dataset can be accessed at https://www.ncbi.nlm.nih.gov/sra/PRJNA769204 (accessed on 7 October 2021).

A total of 4070 genes were differentially expressed in PR-C_2_H_4_ vs. PR-H_2_O (treatment with ethylene and water). A total of 1835 and 2235 genes were regulated and down-regulated in primordia treated with ethylene compared to water, respectively (Figure 4B). All differentially expressed genes were analyzed using KEGG pathway classification. The findings indicate that the differentially expressed genes were primarily involved in carbohydrate and amino acid metabolism (Figure 4C). Additionally, the Gene Ontology classification was used to examine all differentially expressed genes. The results indicate that up-regulated genes were involved in biological regulation, cellular component organization or biogenesis, locomotion, regulation of biological processes, the macromolecular complex, nucleoids, antioxidant activity, electron carrier activity, enzyme regulator activity, and structural molecule activity compared with the down-regulated genes (Figure 4D). Moreover, an ethylene treatment disrupted several biological processes and reorganized secondary metabolisms.

### 3.4. Integrated Analysis of Transcriptomics and Metabolomics Data

Metabolomics analysis revealed that some intermediates involved in the tricarboxylic acid (TCA) cycle, multiple amino acid metabolisms, and secondary metabolism were differentially accumulated. For instance, citrate, ornithine, methionine, spermine, and mevalonate-5P were significantly decreased after ethylene treatment according to LC-MS (Figure S3 and S4). However, the transcriptomics analysis results show that ethylene treatment up-regulated the expression of various genes involved in the TCA cycle, multiple amino acid metabolisms, and secondary metabolism. In response to ethylene treatment, there was an up-regulation of gene expression encoding enzymes involved in the alanine, aspartate, and glutamate metabolism (Table S3). The results suggest that ethylene could accelerate normal glucose metabolism, increasing the participation of intermediates in downstream biological processes.

**Figure 5:**
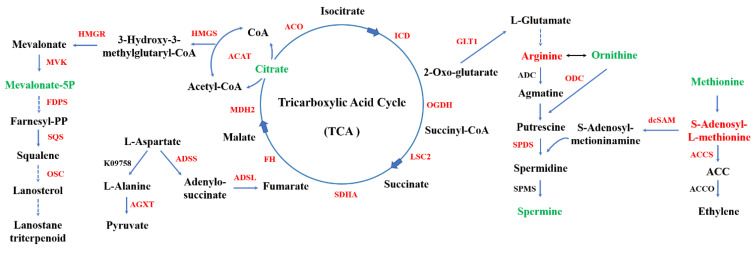
**The expression pattern of genes and metabolites involved in key metabolism pathway**. HMGS, hydroxymethylglutaryl-CoA synthase; HMGR, 3-hydroxy-3-methylglutaryl-coenzyme A reductase; MVK, mevalonate kinase; FDPS, farnesyl diphosphate synthase; SQS, squalene synthase; OSC, lanosterol synthase; ACAT, acetyl-CoA acetyltransferase; AGXT, alanine-glyoxylate transaminase; ADSS, adenylosuccinate synthase; ADSL, adenylosuccinate lyase; ACO, aconitate hydratase; ICD, isocitrate dehydrogenase; OGDH, 2-oxoglutarate dehydrogenase E1 component; LSC2, succinyl-CoA synthetase beta subunit; SDHA, uccinate dehydrogenase (ubiquinone) flavoprotein subunit; FH, fumarate hydratase; MDH2, malate dehydrogenase; GLT1, glutamate synthase; ADC, arginine decarboxylase; ODC, ornithine decarboxylase; SPDS, spermidine synthase; SPMS, Spermine synthase; dcSAM, decarboxylated S-adenosylmethionine; ACC, 1-Aminocyclopropane-1-carboxylate; ACCS, ACC synthase; ACCO, ACC oxidase. The red color indicates up-regulation, green indicates down-regulation, and black indicates not detected.

We examined the encoding enzymes involved in terpenoid backbone biosynthesis to elucidate mechanisms by which the ethylene signal affects triterpenoid biosynthesis. There were seven genes encoding enzymes using transcriptome annotation. The expressions of HMGS (GL24922) and SQS (GL21690) genes were significantly up-regulated when treated with ethylene (*p* < 0.01). This was a two-fold increase compared to the control. The gene expressions of ACAT (GL26574), HMGR (GL24088), MVK (GL17879), FDPS (GL22068), and OSC (GL18675) were slightly up-regulated by 1.37-fold, 1.46-fold, 1.71-fold, 1.05-fold, and 1.33-fold, respectively (Figure 5, Table S3). The results indicate that the ethylene signal up-regulated the expression of genes encoding enzymes involved in terpenoid backbone biosynthesis. These findings suggest that ethylene could help increase the total GA content (Figure 1B).

Indeed, the ethylene production process is enzymatic, mainly via the ACC pathway. The transcriptomics data revealed that ethylene treatment up-regulated the expression of the gene encoding ACCS (GL22580) by 3.30-fold. This supports the hypothesis that exogenous ethylene improves endogenous ethylene accumulation in *G. lucidum*, and this is consistent with the result illustrated in Figure 1A. Additionally, we found that the ethylene signal affected the polyamine metabolism pathway. The genes encoding dcSAM (GL GL17796), ODC (GL17083), and SPDS (GL22111) were up-regulated by 1.74-fold, 1.13-fold, and 3.89-fold, respectively, in the polyamine metabolism pathway. This confirms a previous report that polyamine metabolism could regulate GA production in response to various environments.

## 4. Discussion

This work showed that combining transcriptomics and nontargeted metabolomics analysis provides insights into preliminary studies of physiological alterations at genetic and metabolic levels in *G. lucidum* in response to ethylene. Of note, metabolites are identified based on the accurate mass number, secondary fragments, and isotope distribution, using the Human Metabolome Database, Lipidmaps (V2.3), METLIN databases, and self-built databases. In theory, we used transcriptomics with nontargeted metabolomics data to evaluate the mechanism of global ethylene responses in *G. lucidum*.

### 4.1. Terpenoid Backbone Biosynthesis Pathway

We discovered that ethylene promoted the biosynthesis of secondary metabolites, specifically C27 lanostanes. So far, over 400 secondary metabolites have been isolated from different strains of *Ganoderma* species, with the most abundant being lanostane triterpenoids, including C30 lanostanes (ganoderic acids, aldehydes, alcohols, esters, glycosides, lactones, and ketones), C27 lanostanes, C24, and C25 lanostanes [25]. Lanostane triterpenoids are a class of triterpenoids derived from the terpenoid backbone biosynthetic pathway. The results suggest that ethylene treatment up-regulated the expression of genes encoding enzymes involved in terpenoid backbone biosynthesis. The increased ganoderic acid concentration could be explained by the high expression of related enzymes involved in terpenoid backbone biosynthesis. Nevertheless, several other regulatory mechanisms should be considered. 

### 4.2. Polyamine Metabolism Pathway

Interestingly, the findings indicate that polyamine metabolism might play a role in regulating ganoderic acid biosynthesis. Indeed, many organisms, including plants and fungi, establish novel cellular polyamine homeostasis by adjusting the concentrations of protective metabolites (putrescine, spermidine, and spermine) [26]. Polyamine metabolism responds to many environmental stresses, including heat stress, by regulating reactive oxygen species (ROS) homeostasis [27] and mediating various downstream secondary metabolisms [28]. Under the heat stress (HS), it was firstly found that ROS affected the biosynthesis of GAs [27]. Subsequently, Wu found that ODC-mediated production of putrescine regulates intracellular ROS levels, which influences the gene expression of key enzymes and GA biosynthesis [29]. Recently, it found that spermidine plays a more predominant and stimulative role than Put under HS [30]. Collectively, the polyamine metabolism plays important role in stress responses and GA biosynthesis.

In this study, we found that the polyamine metabolism pathway is involved in the ethylene response. Previous studies suggest that intermediate metabolites or enzymes involved in the polyamine metabolism pathway could regulate ganoderic acid biosynthesis [29,30]. However, the molecular mechanism by which polyamine is regulated remains unknown. 

### 4.3. Ethylene Signaling Pathway

It is puzzling that no typical ethylene-responsive element-binding factors (ERFs) have been identified in the genomes of macrofungi. To initiate the ethylene response, ethylene must be detected and transduced via a signal transduction pathway [31,32]. ERFs are important transcription factors in the ethylene signaling pathway in *Arabidopsis thaliana* [33,34], and the *AtERF* genes are differentially regulated by ethylene. In this study, we found that *G. lucidum* responds to the ethylene signal and improves the contents of secondary metabolites. The findings imply that *G. lucidum* possesses an ethylene signaling pathway. To date, we have identified one homeobox transcription factor gene (*GL25472*) with an ethylene-responsive *cis*-acting element referred to as the GCC box in the promotor sequence. The GCC box is a highly conserved DNA-binding domain unique to ERFs [35]. It operates as a transcriptional activator or repressor in response to ethylene signal regulating the downstream expression of genes. 

On the other hand, homeobox transcription factors play an important role in the secondary metabolic pathway. Cary reported the homeobox transcription factor gene *hbx1* as a regulator of aflatoxin biosynthesis in *Aspergillus flavus* [36]. Thus, it is conceivably hypothesized that homeobox transcription factors have a role in responding to the ethylene signal and regulating secondary metabolism in fungi. However, the biological functions of fungal ethylene response factors are unknown and require additional research. In future research, novel measures to aid in the improvement of secondary metabolites may be developed once the regulatory mechanism of the biosynthesis of the secondary metabolite in *Ganoderma* is unraveled.

## 5. Conclusions

In conclusion, ethylene treatment significantly increases the GA content of *G. lucidum*. The transcriptomics and nontargeted metabolomics data revealed that ethylene treatment significantly up-regulated the expression of genes involved in the TCA cycle, polyamine metabolism pathway, ACC pathway, and secondary metabolism, whereas some important intermedia metabolites were decreased. In this study, we found the main metabolism pathways involved in responding to ethylene signals. The findings will contribute significantly to our understanding of secondary metabolites biosynthesis in fungi. However, the regulatory mechanism of polyamine metabolism and other pathways are still unclear. In the future, we will study the regulatory network associated with the ethylene signaling pathway.

## Data Availability

Any data or material that support the findings of this study can be made available by the corresponding author upon request.

## Supplementary Materials

The following supporting information can be downloaded at: https://www.mdpi.com/article/10.3390/jof8050456/s1. Figure S1: The heatmap of the top 20 differentiated metabolites in *Ganoderma lucidum*. Figure S2: The heatmap of ganoderic acids in *Ganoderma lucidum*. Figure S3: The expression pattern of metabolites involved in the metabolic pathway. Figure S4: The HPLC chromatograms of metabolites involved in the metabolic pathway. Table S1: The differentially accumulated metabolites with ethylene treatment. Table S2: The differentially accumulated ganoderic acid with ethylene treatment. Table S3: The differentially expressed genes involved in the pathway.
