# Supplementary material for: Integrated Transcriptomics and Nontargeted Metabolomics Analysis Reveal Key Metabolic Pathways in Ganoderma lucidum in Response to Ethylene
Source: J Fungi (Basel). 2022 Apr 28;8(5):456. doi: 10.3390/jof8050456 (PMC9146657; doi:10.3390/jof8050456)
Supplement: Supplementary file 1 [file jof-08-00456-s001.zip › Supplementary Materials.pdf]

Supplementary materials:

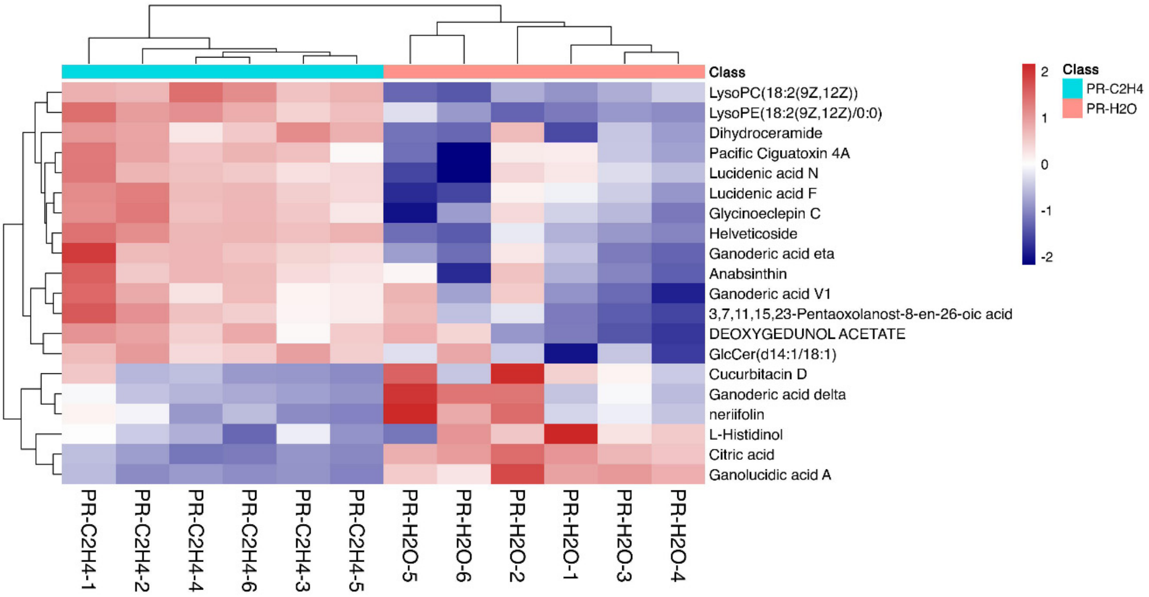

Figure S1 The heatmap of the top 20 differentiated metabolites in *Ganoderma lucidum*

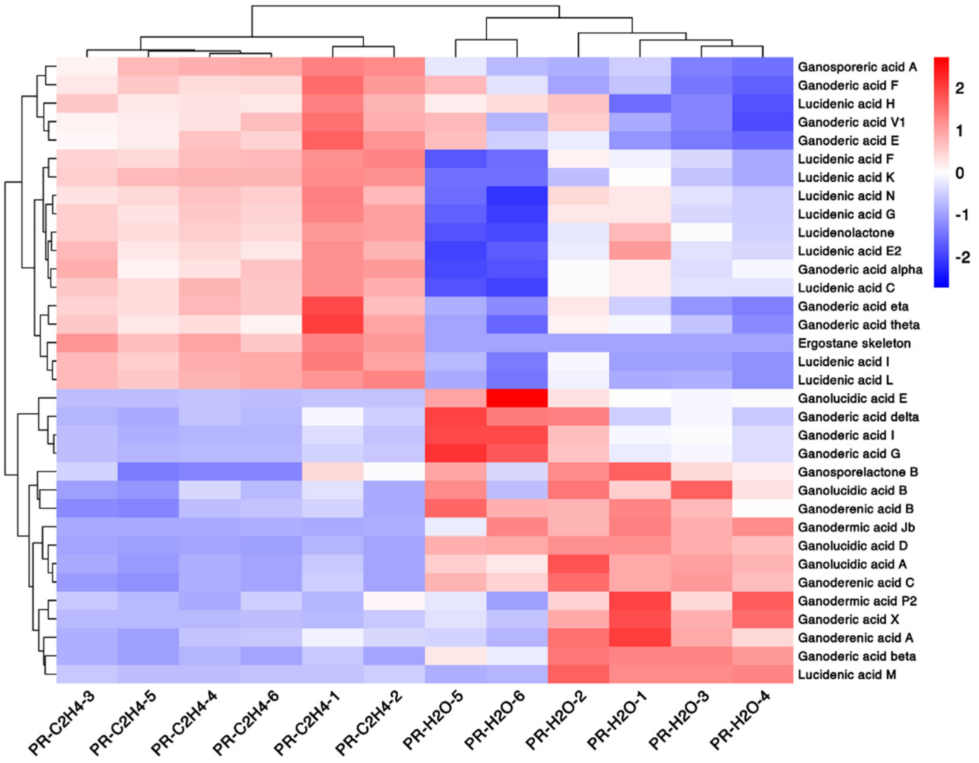

Figure S2 The heatmap of ganoderic acids in *Ganoderma lucidum*

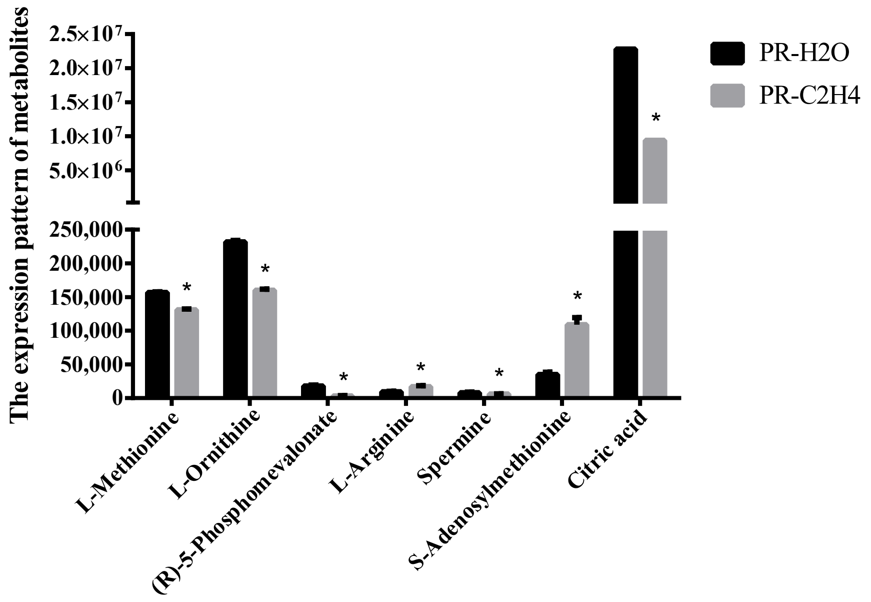

Figure S3 The expression pattern of metabolites involved in the metabolic pathway

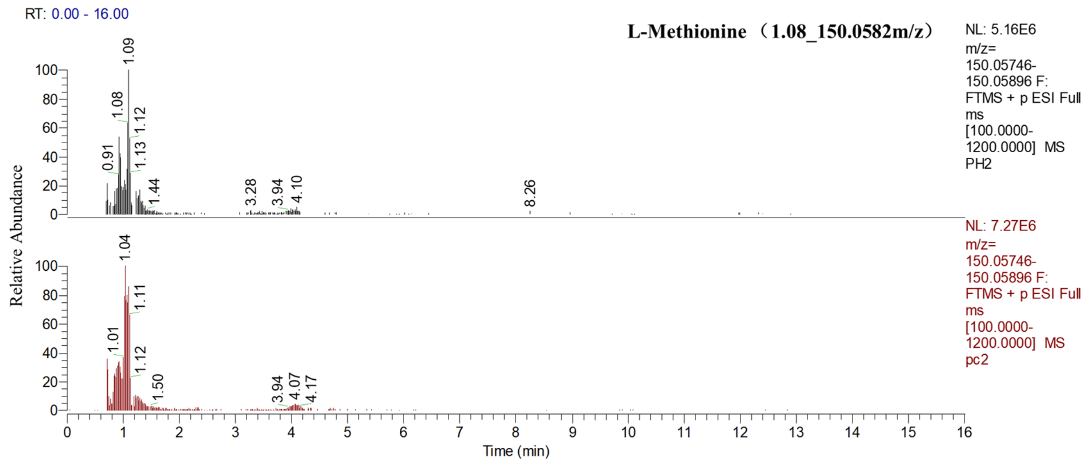

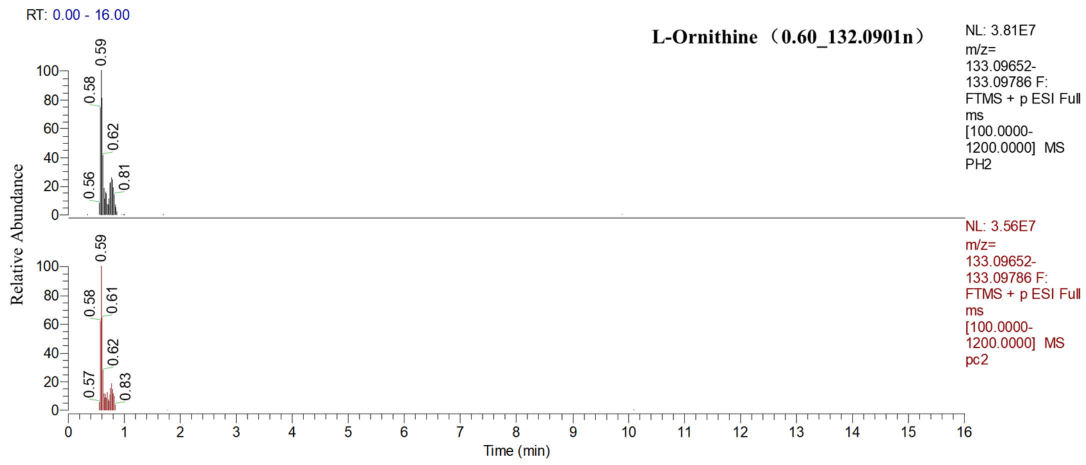

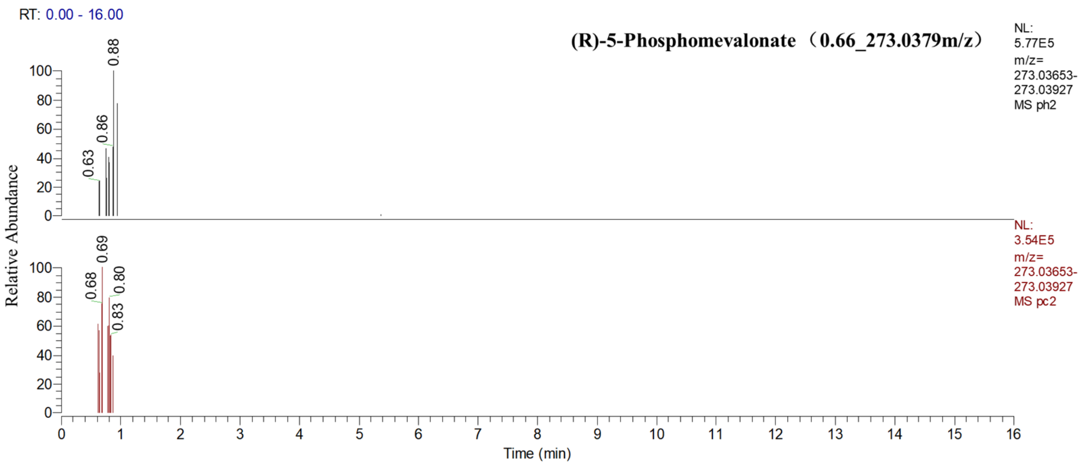
RT: 0.00 - 16.00
(R)-5-Phosphomevalonate（0.66_273.0379m/z）
NL: 5.77E5 m/z= 273.03653-273.03927 MS ph2
NL: 3.54E5 m/z= 273.03653-273.03927 MS pc2
Relative Abundance
Time (min)

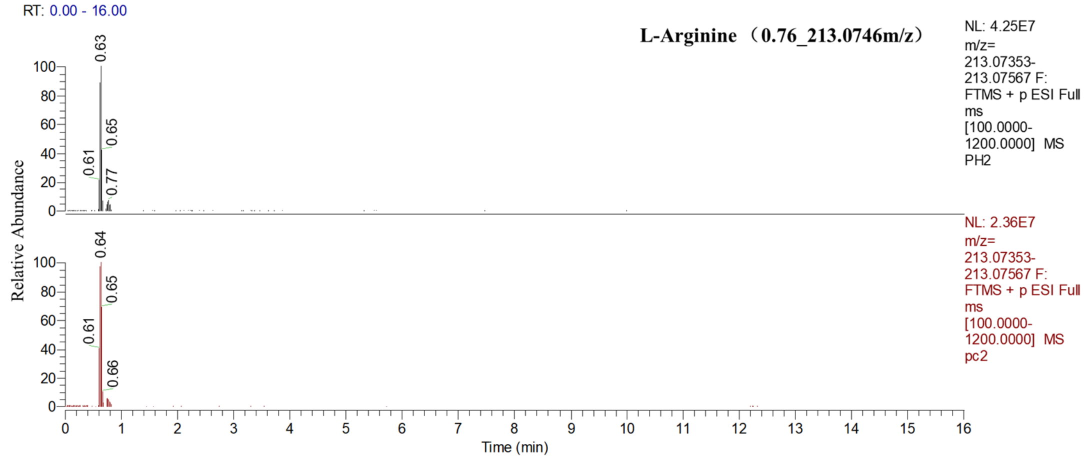
RT: 0.00 - 16.00
L-Arginine（0.76_213.0746m/z）
NL: 4.25E7 m/z= 213.07353-213.07567 F: FTMS + p ESI Full ms [100.0000-1200.0000] MS PH2
NL: 2.36E7 m/z= 213.07353-213.07567 F: FTMS + p ESI Full ms [100.0000-1200.0000] MS pc2
Relative Abundance
Time (min)

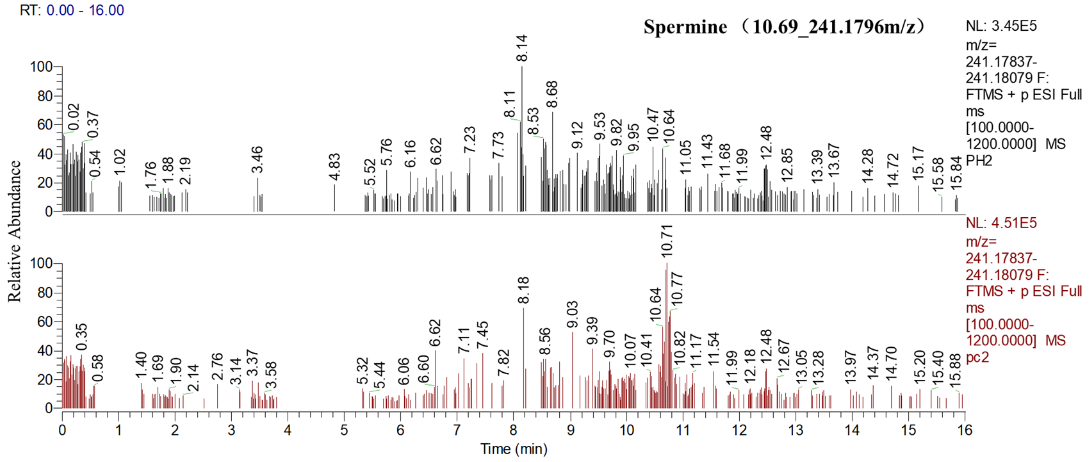
RT: 0.00 - 16.00
Spermine（10.69_241.1796m/z）
NL: 3.45E5 m/z= 241.17837-241.18079 F: FTMS + p ESI Full ms [100.0000-1200.0000] MS PH2
NL: 4.51E5 m/z= 241.17837-241.18079 F: FTMS + p ESI Full ms [100.0000-1200.0000] MS pc2
Relative Abundance
Time (min)

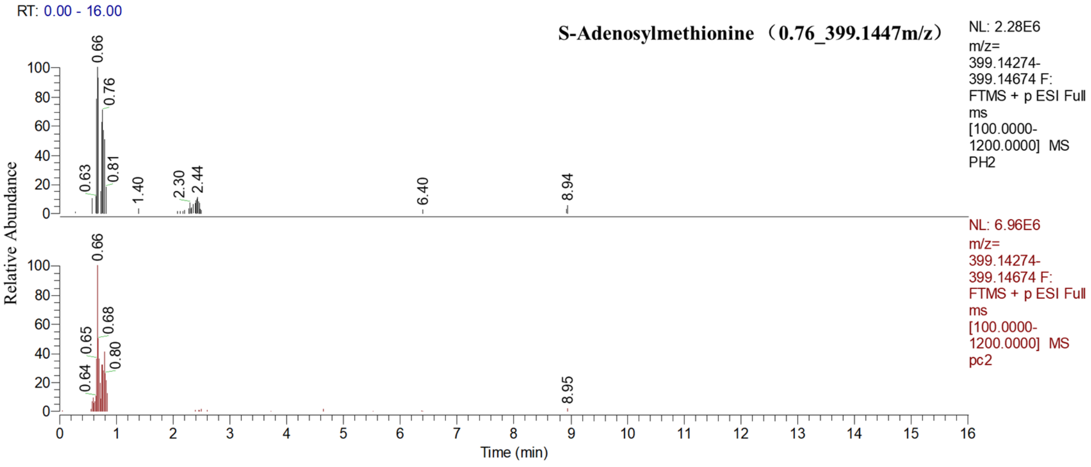
RT: 0.00 - 16.00
S-Adenosylmethionine（0.76_399.1447m/z）
NL: 2.28E6 m/z= 399.14274-399.14674 F: FTMS + p ESI Full ms [100.0000-1200.0000] MS PH2
NL: 6.96E6 m/z= 399.14274-399.14674 F: FTMS + p ESI Full ms [100.0000-1200.0000] MS pc2
Relative Abundance
Time (min)

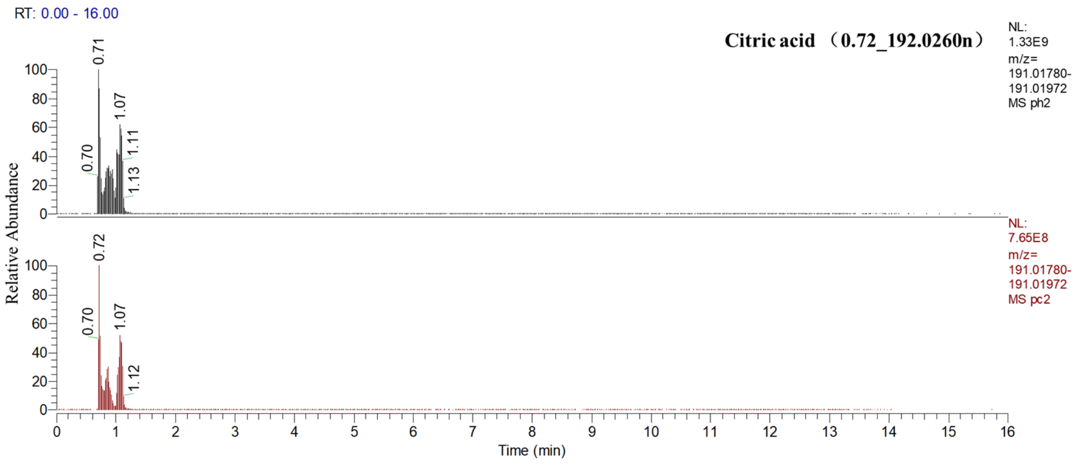

Figure S4 The HPLC chromatograms of metabolites involved in the metabolic pathway
